# Gene expression markers of tendon fibroblasts in normal and diseased tissue compared to monolayer and three dimensional culture systems

**DOI:** 10.1186/1471-2474-10-27

**Published:** 2009-02-26

**Authors:** Sarah E Taylor, Anne Vaughan-Thomas, Dylan N Clements, Gina Pinchbeck, Lisa C Macrory, Roger KW Smith, Peter D Clegg

**Affiliations:** 1 Department of Veterinary Clinical Science, University of Liverpool, Neston, South Wirral, CH64 7TE, UK; 2 Royal (Dick) School of Veterinary Studies, University of Edinburgh, Easter Bush Veterinary Centre, Roslin, EH25 9RG, UK; 3 Royal Veterinary College, Department of Veterinary Clinical Science, North Mymms, Northampton, UK

## Abstract

**Background:**

There is a paucity of data regarding molecular markers that identify the phenotype of the tendon cell. This study aims to quantify gene expression markers that distinguish between tendon fibroblasts and other mesenchymal cells which may be used to investigate tenogenesis.

**Methods:**

Expression levels for 12 genes representative of musculoskeletal tissues, including the proposed tendon progenitor marker scleraxis, relative to validated reference genes, were evaluated in matched samples of equine tendon (harvested from the superficial digital flexor tendon), cartilage and bone using quantitative PCR (qPCR). Expression levels of genes associated with tendon phenotype were then evaluated in healthy, including developmental, and diseased equine tendon tissue and in tendon fibroblasts maintained in both monolayer culture and in three dimensional (3D) collagen gels.

**Results:**

Significantly increased expression of scleraxis was found in tendon compared with bone (P = 0.002) but not compared to cartilage. High levels of COL1A2 and scleraxis and low levels of tenascin-C were found to be most representative of adult tensional tendon phenotype. While, relative expression of scleraxis in developing mid-gestational tendon or in acute or chronically diseased tendon did not differ significantly from normal adult tendon, tenascin-C message was significantly upregulated in acutely injured equine tendon (P = 0.001). Relative scleraxis gene expression levels in tendon cell monolayer and 3D cultures were significantly lower than in normal adult tendon (P = 0.002, P = 0.02 respectively).

**Conclusion:**

The findings of this study indicate that high expression of both COL1A2 and scleraxis, and low expression of tenascin-C is representative of a tensional tendon phenotype. The *in vitro *culture methods used in these experiments however, may not recapitulate the phenotype of normal tensional tendon fibroblasts in tissues as evidenced by gene expression.

## Background

Tendon injuries are a significant cause of morbidity in both man and veterinary species and are reported to represent 30% of the musculoskeletal caseload in a one year study of human general practitioners [1]. Injury to the equine superficial digital flexor tendon (SDFT) poses a significant problem amongst racing Thoroughbreds with a reported incidence of 11–43% [2,3]. The extracellular matrix (ECM) of flexor tendons has evolved not only to both transmit forces from muscle to bone but also as an elastic energy store for efficient locomotion [4]. However, once a tendon has been injured a fibrous repair response ensues which, whilst being very efficient at repairing the damaged tissue, does not regenerate the original tendon matrix [5]. The fibrous scar tissue does not recapitulate the unique parallel collagen fibre alignment found in normal tendon. Consequently, the healed tendon does not retain the biomechanical properties of the original tendon prior to injury [5] and re-injury rates in horses can be as high as 56% [6].

The poor clinical outcome associated with tendon injury and the limited capacity for regeneration of injured tendon have resulted in a growing interest in the use of tissue engineering approaches for tendon therapy in both man and animals [7]. Objective demonstration of successful regeneration requires the identification of markers of tenogenesis. However, currently there are no specific molecular markers that can be used to characterise tendon fibroblasts [8] and identify relevant differentiation and repair. Verification of the success of tendon tissue engineering interventions is currently based on histological analysis and mechanical testing [9]. Identification of pertinent gene expression would be beneficial in confirming cell differentiation to a relevant tendon cell phenotype. Expression of key matrix genes within tissue engineered constructs has also been used to identify such differentiation, although validation of the relevance of the candidate marker genes has not yet been performed [10]. Unfortunately many of these key matrix genes are expressed in a variety of mesenchymal tissues and therefore may not be sufficiently discriminatory. In contrast, the genes COL2A1, COL10A1 and SOX9 are well accepted as being representative of chondrogenic differentiation [11] and Runx2 and osteopontin are discriminating for osteogenic differentiation [11]. In the current study eleven genes were selected as representative of tendon, cartilage and bone and quantified in these musculoskeletal tissues to identify which of these genes were most discriminating for a tendon phenotype.

Collagen type I forms 95% of the collagen content of normal adult tendons, the remaining 5% constitutes small amounts of collagen types III, V, VI, XII and XIV [12]. Following acute rupture of the human Achilles tendon or equine SDFT gene expression of collagen types I, III and V is increased [13-16]. Tenascin-C is also up regulated following tissue wounding [17] and in degenerate tendinopathy [18]. Other important tendon matrix components include the small leucine rich proteoglycans (lumican, decorin, biglycan and fibromodulin). The role of these smaller matrix components has been demonstrated to be important in collagen fibrillogenesis [19]. In addition the glycoproteins such as cartilage oligomeric matrix protein (COMP) and tenascin-C are understood to be involved in collagen fibrillogenesis [20-22].

COMP is an extracellular matrix glycoprotein that is abundant in tissues subjected to load. Levels of COMP increase until skeletal maturity is reached after which time levels gradually decline [20]. Tenomodulin is a type II transmembrane glycoprotein that is preferentially expressed in dense connective tissues. Mice lacking tenomodulin have tendons with a disrupted fibril structure and exhibit severely reduce tendon fibroblast proliferation [23]. Tenomodulin has been reported to be a good phenotype marker for tendon fibroblasts [24]. Decorin is a member of the SLRPs that have been shown to be important in control of collagen fibrillogenesis [25]. Recent investigations have highlighted scleraxis as a specific marker of tendon progenitor cells [26-28]. Furthermore, scleraxis null mutant mice have distinct tendon defects [26]. Runx2 is an osteogenic transcription factor [11]. Osteopontin is a glycoprotein found in bone and forms part of the inorganic component of bone. Osteomodulin is a keratan sulphate proteoglycan found in the ECM of bone and thought to be a marker of osteoblast maturation [29]. SOX9 is a transcription factor important in chondrocyte regulation and is frequently used to identify chondrogenic differentiation [11].

The study performed here was designed to identify the presence of the eleven genes in normal tendon and quantify their expression levels in tendon compared to other musculoskeletal tissues. The expression of key genes which could identify an adult tendon phenotype were then characterised both in clinical cases of tendinopathy of the equine SDFT, as well as during development. Furthermore the expression profile of these genes was characterised *in-vitro *in both monolayer and three dimensional (3D) cultures of tendon fibroblasts to identify whether such models fully recapitulate the tendon phenotype.

## Methods

### Tissue samples

Tissue samples were obtained from animals subjected to euthanasia for clinical reasons other than orthopaedic disease. Full informed written consent was obtained from animal owners for all tissue collection and tissue collection was subject to ethical review. Three samples (10 × 5 × 2 mm) of equine superficial digital flexor tendon were obtained from the mid-metacarpal region from skeletally mature (aged 4–10 years) horses free of clinical orthopaedic disease. Matched cartilage samples (10 × 3 × 0.5 mm) were harvested from the articular surface of the distal aspect of the third metacarpal bone of the same donors with bone (10 × 5 × 2 mm) being harvested from the distal metacarpal epiphysis. Further samples were collected from five skeletally immature animals (three mid-gestational foetuses and two yearlings). All tissue specimens were grossly normal on post mortem examination. Pathological tissue specimens were collected from the mid-metacarpal region of the SDFT of 3 horses with acute (< 6 weeks duration) and 3 horses with chronic (> 6 months duration) tendinopathy. All samples were obtained within 4 hours of euthanasia and collected into RNAlater™ (Ambion, Applied Biosystems). Samples were stored at 4°C for 24 hours and then frozen at -80°C until used for RNA extraction.

### Monolayer cell culture

For the cell culture experiments tendon samples were collected from 4 adult (age 4–10 yrs), mixed breed horses subjected to euthanasia. All tendons were free of pathology on clinical and post mortem examination. Tendons were collected from the SDFT at the level of the mid-metacarpus (tensional), within 4 hours of euthanasia. Samples were cut into 2 × 2 × 2 mm^3 ^pieces and subjected to collagenase type II (0.1%) digestion overnight in DMEM (Sigma) containing 5% FCS, penicillin/streptomycin and amphotericin) on an orbital shaker at 37°C. The isolated tendon fibroblasts were cultured in 75 cm^2 ^flasks in DMEM containing 10% FCS, penicillin/streptomycin and fungizone at 5% CO_2 _and 37°C until approximately 90% confluent before passaging (usually 4–6 days for each passage). Cells were released from flasks with trypsin (0.05%) and re-seeded into flasks at 5000 cells/cm^2^. Culture medium was changed every 3–4 days. One millilitre of Tri-Reagent (Sigma) was added to the remaining cells for subsequent RNA extraction, these samples were stored at -80°C. The cells were passaged 5 times for the 3 different horses.

### Three dimensional cell culture in collagen gels

Tendon fibroblasts harvested from the tensional area of the SDFT as described above were cultured until confluent. Collagen gels were prepared following a method described previously [30]. Briefly, following trypsinisation at the end of passage one, cells were counted and suspended in 90% type I collagen gel (2 mg/ml) (Invitrogen), 10% 10 × DMEM (Sigma) using 1 × 10^6 ^cells/mL. Two hundred microlitres of the collagen cell suspension were cast in a trough mold (Trough loader, Flexcell International) centrally located in a Tissue train culture plate (Flexcell International). The collagen gels were cultured at 5% CO_2 _in a humidified incubator at 37°C for 24 hours prior to the addition of DMEM containing 10% FCS, penicillin/streptomycin and amphotericin. Cell seeded collagen gels were then cultured without dynamic load for 5 days.

### RNA extraction and quantification

RNA was extracted using phenol/guanidine HCl reagents (TriReagent™, Sigma) and isolated using the published methods [31,32]. Tissue samples were pulverised for 1 minute at 2000 oscillations/minute in a liquid nitrogen cooled dismembranator (Braun Mikro-Dismembrator Vessel, Braun Biotech International, Melsungen, Germany). A 1 mL aliquot of phenol/guanidine HCl reagents (TriReagent™, Sigma) was added to the powdered tendon, cartilage or bone. Two hundred microlitres of chloroform was added to the microcentrifuge tubes prior to centrifugation at 12,000 g for 10 minutes. RNA was extracted from the aqueous phase using a commercially available RNA purification kit (RNeasy Mini Kit, Qiagen). Digestion of DNA was carried out using a commercially available kit (RNase-Free DNase Set, Qiagen). RNA was stored at -80°C prior to reverse transcription.

### Reverse transcription

The purified RNAs were measured (ND-1000 spectrophotometer Nanodrop Technologies) and then 1 μg RNA was used to prepare cDNA using M-MLV reverse transcriptase and random primers according to the manufacturer's instructions (Promega). Samples were stored at -20°C prior to relative quantification of gene expression.

### Primer Design

Transcript sequences were obtained from the National Centre for Biotechnology Information (Bethesda, MD, USA) (Tables 1 &2). Equine gene sequences were aligned to human, bovine and canine sequences using online software (, ) to predict exon boundaries. Primers were designed using Primer Express (Applied Biosystems) software and selected to span predicted exon boundaries where possible. BLAST searches were performed for all sequences to confirm gene specificity. Target and reference gene primers were synthesized by Eurogentec. All primers were validated using a standard curve of five serial dilutions so that all primer efficiencies were between 95–105% (Tables 1 and 2).

**Table 1:** Reference gene primer sequences used for qPCR

Gene	SCS	Efficiency (%)	Sequence
GapDH AF157626	-3.32	100.2	5'GCATCGTGGAGGGACTCA3' 3'GCCACATCTTCCCAGAGG5'
18S AJ311673	-3.24	103.3	5'GGCGTCCCCCAACTTCTTA3' 3'GGGCATCACAGACCTGTTATTG5'
ACTB AF035774	-3.33	99.9	(Bogaerts et al., 2006) 5'CCAGCACGATGAAGATCAAG3' 3'GTGGACAATGAGGCCAGAAT5'
SDHA DQ402987	-3.29	101.5	5'ACAGAGGAATGGTCTGGAATACTGA3' 3' GTGAGCACCACGTGACTCCTT5'

SCS = Standard Curve Slope

**Table 2:** Target gene primer sequences use for qPCR

Gene	SCS	Efficiency (%)	Sequence
COL1A2 XM_001492962	-3.31	100.5	5'GCACATGCCGTGACTTGAGA3' 3'CATCCATAGTGCATCCTTGATTAGG5'
COL2A1 NM_001081764	-3.21	105.0	5'TCAAGTCCCTCAACAACCAGATC3' 3'GTCAATCCAGTAGTCTCCGCTCTT5'
COL3A1 XM_001501719	-3.22	104.6	5'ACGCAAGGCCGTGAGACTA3' 3'TGATCAGGACCACCAACATCA5'
COL10A1 XM_001504101	-3.39	97.4	5'TGCCCAGTGGACAGGTTTCT 3' 3'GTCTTTTCGTTTCTAGTCAGATTTTGAA5'
COMP AF325902	-3.42	95.7	5'GGTGCGGCTGCTATGGAA3' 3'CCAGCTCAGGGCCCTCAT5'
DCN AB106279	-3.31	100.1	5'CATCCAGGTTGTCTACCTTCATAACA3' 3'CCAGGTGGGCAGAAGTCATT5'
TNC XR_035757	-3.36	98.6	5'GGGCGGCCTGGAAATG3' 3'CAGGCTCTAACTCCTGGATGATG5'
TMD AB059407	-3.38	97.4	5'ACGTGACCATGTATTGGATCAATC3' 3'CACCATCCTCCTCAAAGTCTTGT5'
SIX1 XM_001492786	-3.23	104.0	5' GATGCCCCAATGTTTGTGATG3' 3' AGGAGGCATTGCTGACAATCTT5'
SCXB NM_001105150	-3.35	98.8	5'TCTGCCTCAGCAACCAGAGA3' 3'TCCGAATCGCCGTCTTTC5'
SOX9 XM_001498424	-3.33	99.9	5' CTTTGGTTTGTGTTCGTGTTTTGT3' 3'AGAGAAAGAAAAAGGGAAAGGTAA GTTT5'
OPN XM_001496152	-3.33	99.9	5'CGCAGATCTGAAGACCAGTATCCT3' 3'TGCTTTCCACAGGTGATGTGA5'
RUNX2 XM_001502519	-3.36	98.4	5'CTGGGCCATGTGTATGATTTGT3' 3'TTTTGACCTGATATAGAGTGCATGGT5'
OMD XR_035840	-3.26	103.4	5'CAAATTCATCAACCCCTGAAA3' 3'CTTCATCTGGCTCTTGGTCA5'

### Normalisation of reference genes

The most stable pair of reference gene primers were selected using the normalization strategy proposed by [33]. Verification of reference gene selection was carried out using Normfinder [34]. Reference gene stability packages were downloaded from geNorm:  and Normfinder: .

### RT-PCR

Real time RT (qPCR) assays were performed in triplicate using the 7900 HT Fast Real-Time PCR System (Applied Biosystems; Warrington, UK) in 384 well plates. Reaction volume in each well was 10 μl (4.6 μl of cDNA, 5 μl of Power SYBR mastermix (Applied Biosystems), 0.1 μl of DEPC water 0.15 μl of 3 μM forward primer and 0.15 μl of 3 μM reverse primer). The cycling conditions comprised 10 min polymerase activation at 95°C and 40 cycles at 95°C for 15 sec and 60°C for 60 sec. Data was then analysed using Sequence Detection Systems Software v2.2.1 (Applied Biosystems; Warrington, UK).

### Statistical analyses

All data were presented as mean ± SE. All data departed from normality and were therefore log_10 _transformed. Significant differences in gene expression between cartilage, tendon and bone matched samples were identified using a mixed effects linear regression model to allow for the clustering of samples within individual horses (S-Plus software). Comparisons between gene expression of normal tissue and developing or diseased or cultured cells were carried out using a two sample student's t-test where log transformed data were normally distributed. The level of significance was set at P < 0.05.

## Results

### Normalisation of reference genes

GeNorm and Normfinder identified Glyceraldehyde -3-phosphate dehydrogenase (GapDH) and succinate dehydrogenase subunit A (SDHA) to be the two most stably expressed reference genes across normal tendon, cartilage and bone samples, and both the 2D and 3D *in vitro *cultures (M = 0.045, V = 0.027). The most stably expressed reference genes in tendon development and disease were beta actin (ACTB) and GapDH (M = 0.056, V = 0.057) (Table 3). When all samples were considered together GapDH was identified as the most stable gene (Normfinder ratio 0.050) and ACTB the second most stable gene (Normfinder ratio 0.072) (Table 3). GeNorm identified GapDH and ACTB to be the most stable pair of reference genes (M = 0.127, V = 0.048) (Table 3). As geNorm and Normfinder were in agreement, gene expression data was normalised to the average expression of GapDH and ACTB. The average Ct values used to identify the most stable reference genes can be seen in Additional file 1.

**Table 3:** M and V values generated by geNorm and Normfinder

Sample	Reference Genes	M Value (Gene stability)	V Value (Pairwise stability)	Normfinder
Tissue	GapDH SDHA	0.045	0.027	0.012 0.018
Developing Tissue	GapDH ACTB	0.031	0.020	0.011 0.032
Diseased Tissue	ACTB GapDH	0.056	0.057	0.019 0.068
Monolayer Culture	GapDH SDHA	0.059	0.085	0.020 0.062
Collagen Gel	GapDH SDHA	0.017	0.013	0.006 0.020
All Samples	GapDH ACTB	0.127	0.048	0.050 0.072

Coefficients for tissue samples (tendon, cartilage and bone), developing tendon, diseased tendon, monolayer culture and 3D collagen gels.

## Tissue samples

### Adult mesenchymal tissues

**Figure 1:**
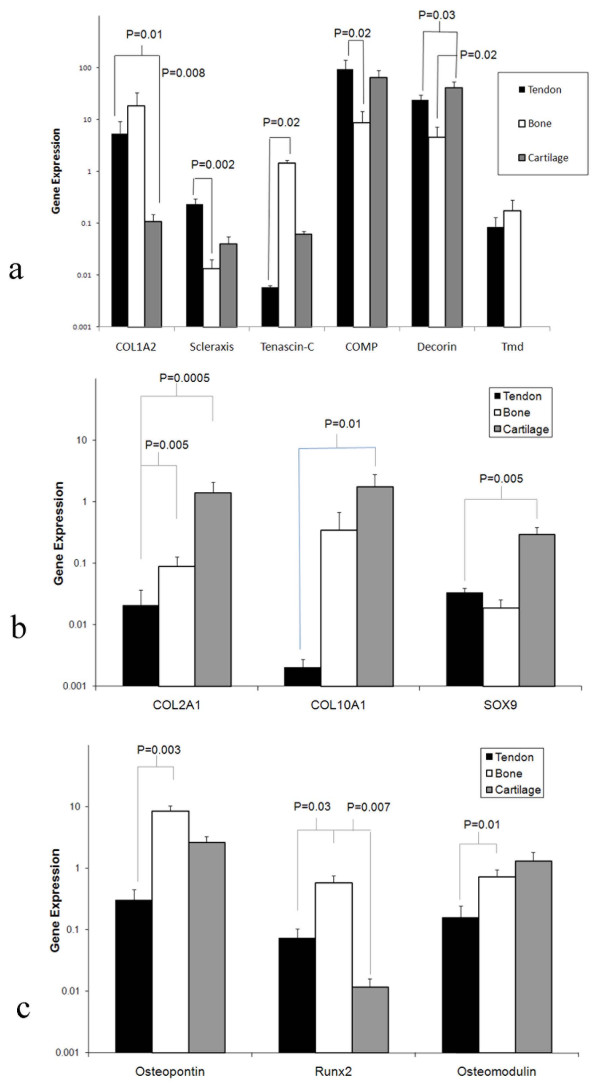
**Gene expression of normal tendon, bone and cartilage samples**. (a) Gene expression of COL1A2, scleraxis, tenascin-C, COMP and decorin in matched samples of tendon, cartilage and bone harvested from normal adult horses. (b) Gene expression of COL2A1, COL10A1 and SOX9 in matched samples of tendon, cartilage and bone harvested from normal adult horses. Relative gene expression data is represented graphically as log transformed values. P values were generated using a mixed effects linear regression model to allow for clustering within individual donors. (c) Gene expression of osteopontin, osteonectin and osteomodulin in matched samples of tendon, cartilage and bone harvested from normal adult horses.

Figure 1 shows the data obtained for expression levels of genes presumed to be associated with tendon (a), bone (b) and cartilage (c). Scleraxis showed significantly higher expression in tendon than in bone (P = 0.002) (Figure. 1a), and whilst higher levels of expression were identified in tendon than cartilage this did not reach statistical significance. As expected, significantly higher COL1A2 was expressed in both tendon and bone than in cartilage (P = 0.01 and P = 0.008, respectively) (Figure 1a). Tenomodulin was identified in both tendon and bone but could not be detected in cartilage. No significant difference could be identified in the levels tenomodulin expression in tendon and bone. Tenascin-C expression was significantly lower in tendon than in bone (P = 0.02). COMP expression was significantly higher in tendon than in bone (P = 0.02) but no difference in expression was identified between tendon and cartilage (Figure. 1a). Decorin expression was highest in cartilage (P = 0.03) and lowest in bone (P = 0.02). Osteopontin, Runx2 and osteomodulin were able to discriminate between tendon and bone (P = 0.003, P = 0.03 and P = 0.01, respectively) (Figure. 1b) but Runx2 was the only hypothesised bone marker that distinguished between bone and cartilage (P = 0.007). Of the cartilaginous markers, only COL2A1 was significantly lower in tendon than in both bone and cartilage (P = 0.005 and P = 0.0003). Cartilage showed higher expression of COL10A1 and SOX9 than tendon (P = 0.01 and P = 0.005 respectively) (Figure. 1c). From the matched normal tissue samples a panel of COL1A2, scleraxis and tenascin-C were selected as the most discriminating genes of tendon phenotype.

### Tendon development

**Figure 2:**
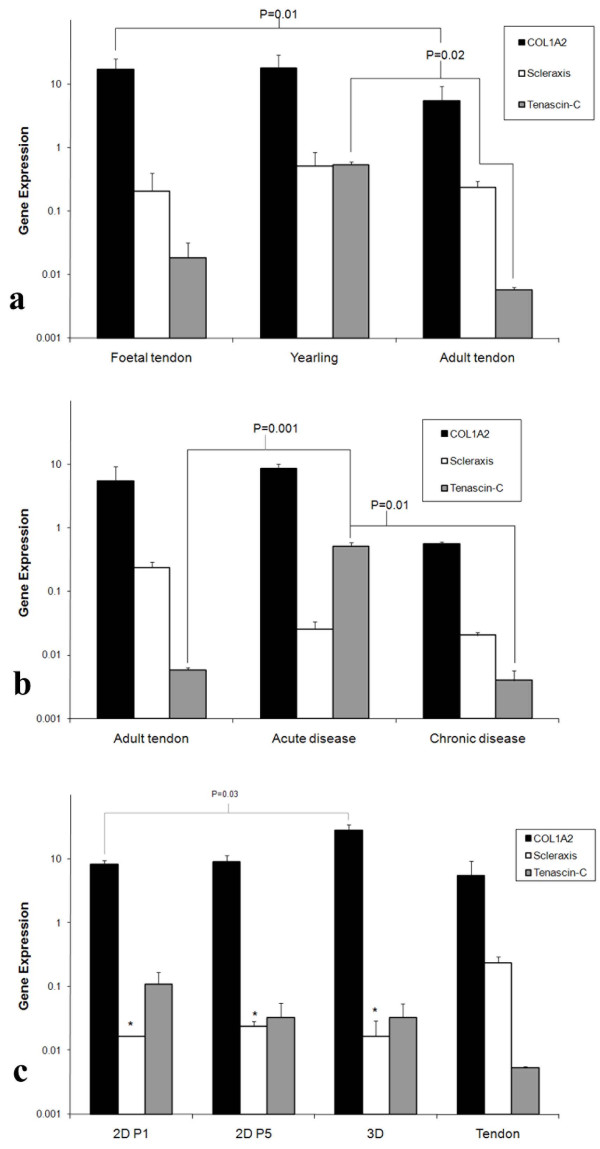
**Gene expression of developing and diseased tendon and *in vitro gene expression***. (a) Gene expression of COL1A2, scleraxis, tenascin-C in SDFT collected from foetuses, yearlings and adults. (b) Gene expression of COL1A2, scleraxis, tenascin-C in acute and chronic disease in comparison to normal adult tendon. (c) Gene expression of COL1A2, scleraxis, tenascin-C in 2D and 3D *in vitro *culture in comparison to normal adult tendon. (2D P1 = passage 1 monolayer culture, 2D P5 = passage 5 monolayer culture, 3D = tendon fibroblasts cultured in collagen gels). P values were generated using a two sample student's t-test for normally distributed log transformed data.

Expression of COL1A2 was higher in the foetal tissues compared to mature tendon relative to reference gene mRNA (P = 0.01). There was increased tenascin-C expression in skeletally immature tendon in comparison to adult tendon (P = 0.02) (Figure. 2a).

### Tendon disease

Acute tendinopathy produced significant changes in gene expression. Tenascin-C expression was greatly increased in acutely diseased tendon in comparison to normal tendon (P = 0.0001), although expression of scleraxis and COL1A1 did not vary. Chronic tendinopathy resulted in a gene expression profile similar to that of normal tissue with no significant difference in the expression of tenascin-C (Figure. 2b).

## Cell culture samples

### Monolayer cell culture

Scleraxis gene expression was decreased compared to normal adult tendon at passage 1 of monolayer culture of tendon fibroblasts and this significant decrease was maintained at passage 5 (P = 0.002 and P = 0.004 respectively) (Figure. 2c). No significant differences in COL1A2 and tenascin-C expression were identified between normal tissue and the 2D *in vitro *culture systems. Tenomodulin gene expression levels were not detectable in the *in vitro *cells.

### Three dimensional cell culture in collagen gels

Placing tendon fibroblasts into a three dimensional collagen matrix resulted in increased expression of type I collagen (P = 0.03) compared to passage one monolayer cells. No significant difference was identified between levels of COL1A2 and tenascin-C expression by 3D cultured tendon fibroblasts and normal adult tendon. However, 3D cultured tendon fibroblasts showed significantly reduced expression of scleraxis (P = 0.03) in comparison to normal adult tendon (Figure 2c).

## Discussion

This study has confirmed that a panel of 'marker' genes are required to identify tendon cell phenotype from other mesenchymal tissues. The matched adult samples of tendon, cartilage and bone show the tensional SDFT to express high levels of COL1A2 and scleraxis and low levels of tenascin-C in comparison to the other tissues. Bone also expresses high levels of COL1A2 but contrastingly low levels of scleraxis and high levels of tenascin-C. In contrast, cartilage expresses moderate amounts of COL1A2, scleraxis and tenascin-C. Tendon regions with a fibrocartilagenous phenotype that are subjected to compressive loads have been shown to have to have higher levels of tenascin-C [35-37], for example at the myotendinous and osteotendinous junctions [38]. Low levels of tenascin-C within the mid-body of the normal equine SDFT identified in the current study are likely to be a reflection of the tensile loads placed on this region of the tendon. The presence of scleraxis within equine cartilage may be a consequence of the inclusion of the perichondrium in the samples cartilage, a tissue that is derived from scleraxis expressing cells [39].

Scleraxis has been described as being an important marker of tendon neoformation however, there is currently no evidence that scleraxis can induce tendon neoformation [40]. Murchison et al (2007) clearly demonstrated the importance of scleraxis as a transcription factor during tendon development with scleraxis null mutants exhibiting severe defects in the force transmitting tendons. It has been suggested that scleraxis is important in directing condensations of tendon progenitor cells to form the force transmitting tendons [26]. The current experiments did not identify any significant increase in levels of scleraxis expression in mid-gestational foetal tendons compared to normal adult tendons. It is possible that once the tendon phenotype is established during development there is no further change in scleraxis expression. Alternatively, translational control of the protein may differ between developing and adult tendons. Scleraxis expression showed no significant difference between normal and diseased tendons.

Tendon samples collected from horses with naturally occurring acute tendinopathy of the SDFT did not show a significant increase in COL1A2, this may be a result of the large range in expression levels in the normal adult tendon samples, leading to large standard errors. In man, increased expression of type I collagen has been a consistent finding in acute tendinopathy [41]. Tenascin-C was significantly increased in the acutely diseased samples; this is consistent with the appearance of tenascin-C during the inflammatory phases of wound healing [17,21].

Increased type-I collagen has been reported in chronic degenerate Achilles tendinopathy [42,43] this is at variance with the findings of the current study that identified no significant difference between gene expression of COL1A2 in normal adult tendon and tendinopathy of the SDFT of more than 6 mths duration in the horse. This may reflect differences in clinical condition between painful degenerate Achilles tendons and healed non-painful chronic tendinopathy of the equine SDFT. Levels of tenascin-C mRNA from chronic tendinopathy were not significantly different from normal adult SDFT demonstrating that levels of tenascin-C may only increase transiently in acute injury then to return to normal levels. These findings are at variance with some of the human literature describing degenerate tendons [8,18] where increased tenascin-C may be associated with round cells and a more fibrocartilaginous phenotype or a different disease state.

Monolayer culture of tendon fibroblasts provides a simple method to study cell phenotype. Recent investigations have highlighted significant differences in cellular gene expression in monolayer compared with three dimensional cultures [44]. The gene expression profile of tendon fibroblasts in monolayer culture has recently been shown to alter with progressive passaging [45]. The current experiments have highlighted further differences between monolayer tendon fibroblasts and those found in adult tendon. Scleraxis expression is decreased at both passage one and five in monolayer cultures of tendon fibroblasts hence, neither have a gene expression profile that recapitulates what is found in normal tendon. Three dimensional cell culture methods are thought to more closely mimic the *in vivo *cellular environment [30,44]. Unfortunately whilst increasing the expression of COL1A2 in agreement with the work of others [46] expression of scleraxis was not retained when these cells were cultured in three dimensional collagen gels. The *in vitro *models used in the current study did not fully recreate the adult tendon phenotype. Further work is warranted to identify a culture system that more closely resembles adult tendon. Recently embryonic tendon fibroblasts cultured in a three dimensional fibrin gel [47] were able to maintain a tendon developmental phenotype in the fibrin gel as defined by electron microscopy. Other three dimensional culturing systems have been extensively investigated biomechanically [9] however, the gene expression of these alternative systems has yet to be reported. Clearly, the importance of recreating the transcriptomic profile of normal tendon relative to the functional properties of the engineered tissue requires further evaluation.

The two methods of reference gene stability assessment were in agreement for all experiments carried out in this study, this is concordant with previous findings [48]. Interestingly GapDH was identified as one of the most stable reference genes *in vitro *while Normfinder found ACTB to be the most stable reference gene when comparing normal and diseased tendons.

## Conclusion

As no single molecular marker was capable of discriminating tendon from both bone and cartilage in the current study it is recommended that multiple genes are used to identify tenogenic differentiation. The current experiments would suggest high expression of COL1A2 and scleraxis and low expression of tenascin-C are most representative of the normal adult tendon phenotype. Furthermore, this work refutes the use of tenomodulin as a good marker of equine tendon fibroblasts as similar levels were identified in both tendon and bone. A genome wide screen may in the future identify specific markers of tendon phenotype. Whether they are maintained in cultured fibroblasts will also need to be determined.

## Competing interests

The authors declare that they have no competing interests.

## Authors' contributions

SET carried out the experimental work, participated in the sequence alignment and statistical analyses and drafted the manuscript. LCM participated in the sequence alignment. DNC and AVT participated in reference gene normalisation. GP helped perform statistical analysis. PDC and RKWS conceived the study, and participated in its design and coordination and helped to draft the manuscript. All authors read and approved the final manuscript.

## Pre-publication history

The pre-publication history for this paper can be accessed here:



## Supplementary Material

Additional file 1 **Raw Ct values of reference genes of all samples**. The data provided represent the cycle thresholds of the three reference genes used in the data analysis. Click here for file
