# Supplementary material for: Gene expression markers of tendon fibroblasts in normal and diseased tissue compared to monolayer and three dimensional culture systems
Source: BMC Musculoskelet Disord. 2009 Feb 26;10:27. doi: 10.1186/1471-2474-10-27 (PMC2651848; doi:10.1186/1471-2474-10-27)
Supplement: Additional file 1 — Raw Ct values of reference genes of all samples. The data provided represent the cycle thresholds of the three reference genes used in the data analysis. [file 1471-2474-10-27-S1.docx]

|  | GapDH | ACTB | SDHA |
| --- | --- | --- | --- |
| Tendon2 | 25.20827 | 23.403 | 28.49791 |
| Tendon3 | 20.21815 | 18.31948 | 23.27233 |
| Tendon5 | 22.25185 | 19.6864 | 25.47927 |
| Bone 2 | 26.72089 | 24.98445 | 30.72722 |
| Bone3 | 23.24252 | 21.22666 | 25.931 |
| Bone5 | 20.80887 | 18.36155 | 23.21867 |
| Cartilage 2 | 29.13172 | 29.68464 | 32.70421 |
| Cartilage 3 | 25.51838 | 25.43474 | 28.62801 |
| Cartilage5 | 20.35341 | 19.9389 | 24.27701 |
| FoetalT1 | 18.784 | 17.54 | 20.119 |
| Foetal T2 | 19.806 | 17.779 | 22.705 |
| Yearling1 | 18.498 | 16.51933 | 21.79876 |
| Yearling2 | 18.783 | 16.629 | 21.354 |
| Acute Dz1 | 23.116 | 19.98 | 20.013 |
| Acute Dz 2 | 22.152 | 19.164 | 20.033 |
| Acute Dz 3 | 23.263 | 19.532 | 20.14 |
| Chronic Dz1 | 20.836 | 19.183 | 24.037 |
| Chronic Dz2 | 20.248 | 18.951 | 23.454 |
| Chronic Dz3 | 21.384 | 19.81 | 24.68 |
| P1T1 | 18.705 | 16.109 | 23.52 |
| P1T2 | 21.121 | 28.443 | 25.175 |
| P1T3 | 18.453 | 15.979 | 23.252 |
| P5T1 | 22.548 | 20.576 | 27.563 |
| P5T2 | 26.935 | 24.326 | 31.176 |
| P5T3 | 24.565 | 23.824 | 31.748 |
| Tissue Train1 | 17.865 | 16.452 | 22.265 |
| Tissue Train2 | 17.992 | 16.297 | 23.132 |

Table 4
